# Economic benefits of aquatic invasive species management in freshwater systems: A contingent valuation study

**DOI:** 10.1371/journal.pone.0346515

**Published:** 2026-09-16

**Authors:** Roshan Puri, Lucia R. Levers, Amit K. Pradhananga

**Affiliations:** 1 Center for Watershed Sciences, University of California, Davis, California, United States of America; 2 Sustainable Agricultural Water Systems Research, USDA-ARS, Davis, California, United States of America; 3 Department of Forest Resources, Center for Changing Landscapes, University of Minnesota, St. Paul, Minnesota, United States of America; National Research and Innovation Agency, INDONESIA

## Abstract

Aquatic invasive species (AIS) cause significant ecological and economic impacts, including habitat damage, degradation of water quality, and reduced recreational opportunities. While many of these effects are well documented, less is known about how the general public perceives and engages with AIS in regions with significant freshwater infestations. To this end, we conducted surveys of general residents and lake association members, a self-selected stakeholder sample drawn from a lake advocacy organization in Minnesota, USA, a state where over half of its waters (by area) are affected by AIS, with the objective of estimating households’ willingness to pay (WTP) for managing AIS populations. Using a single-bounded trichotomous-choice question, we asked respondents whether they would support an annual increase in property tax or rent, with the generated revenue earmarked for AIS prevention, control, and containment. We conservatively estimate that general households are willing to pay $35–$40 per year, while lake association members are willing to pay $64–$75 per year. These results indicate broad public support across both samples, with higher WTP among those with direct connections to the affected systems, including recreational users and those who trust resource managers. From a policy perspective, our findings have two implications. First, they can help policymakers and resource managers more effectively target financial and outreach strategies. Second, the WTP estimates provide a foundation for incorporating public preferences into conservation funding decisions.

## 1 Introduction

Aquatic invasive species (AIS) pose significant threats to freshwater ecosystems globally by degrading native habitats, out-competing native species for resources, and undermining essential ecosystem services [1–4]. These threats are especially concerning given the outsized importance of freshwater ecosystems, which support higher species richness per unit area than marine or terrestrial systems and provide disproportionately greater direct benefits to human societies, including for drinking water, food production, recreation, and cultural services [5]. Sadly, these systems are among the most heavily invaded on Earth, due in part to their high connectivity, intensive human use, and limited spatial extent [5]. At present, nearly 40% of freshwater fish species, 70% of freshwater mussels, and over 48% of freshwater crayfish are considered imperiled, with invasive species a leading driver of these declines [6].

Nowhere is this threat more visible than in the Great Lakes region of the United States, home to the state of Minnesota. With over 11,800 lakes and an extensive network of rivers, the state has become increasingly susceptible to the spread of AIS, with recreational boating being one of the main invasion vectors [4]. Currently, approximately 8% of Minnesota’s lakes, representing more than 50% of the state’s total lake surface area [7,8], are known to host at least 37 different AIS [9], including Eurasian watermilfoil (*Myriophyllum spicatum*), curly-leaf pondweed (*Potamogeton crispus*), spiny waterflea (*Bythotrephes longimanus*), common carp (*Cyprinus carpio*), starry stonewort (*Nitellopsis obtusa*), and zebra mussels (*Dreissena polymorpha*).

These widely varying species also have widely varying ecological and economic impacts. Zebra mussels alone are responsible for over $1 billion in damages to industrial and municipal water-intake systems each year in the United States [10], with impacts to drinking water treatment plants, hydropower facilities, industrial water systems, and navigation infrastructure in Minnesota costing hundreds of millions of dollars annually [11,12]. Infestations of Eurasian watermilfoil have been linked to reductions in shoreline property values of up to 16% [13]. Broadly, the cumulative economic impact of biological invasions in the U.S. has been estimated at nearly $123 billion per year, over $423 billion globally, which includes costs to agriculture, aquaculture, recreation, and hydropower [14,15].

Without effective management, invasive species can spread and intensify their impacts rapidly [16]. In response, federal, state, and local agencies already invest millions of dollars annually to manage these threats. For context, federal agencies allocated almost $80 million in 2010 specifically to combat the spread of Asian carp species such as bighead carp (*Hypophthalmichthys nobilis*), black carp (*Mylopharyngodon piceus*), grass carp (*Ctenopharyngodon idella*), and silver carp (*Hypophthalmichthys molitrix*) [12]; Great Lakes states collectively spend about $20 million each year to control the sea lamprey (*Petromyzon marinus*), a parasitic species that preys on native fish populations [12,17]; and the state of Florida dedicates approximately $30 million per year for AIS management within natural areas and state parks [1].

The state of Minnesota has explicitly committed to significantly reduce the spread and impacts of AIS across state waters [18], allocating roughly $20 million annually to this effort, with about $4 million being generated from fees for watercraft registrations and out of state fishing licenses [18–24]. About three-fourths of total spending is focused on boat inspections, including $10 million given directly to counties. The remainder is directed toward admin, coordination, education, management, and almost $1.5 million for research. Starting in 2027, the balance of funding sources is shifting. General funds allocated for AIS will be reduced by $5 million (cutting in half the amount for county boat inspections), while, as of 2026, surcharges for watercraft and out of state licenses have increased [18,25]. The movement towards “pay to play” funding reflects a view that those who enjoy a resource should be the ones to pay to protect it. While boaters and anglers no doubt are impacted by invasive species, it is important to understand that people who have no physical connection to waterbodies may value and be willing to support AIS management as well.

A substantial body of literature has examined public preferences regarding the management of invasive species, with a primary focus on understanding individuals’ knowledge, attitudes, and perceptions [1,2,26–31]. These studies investigate how the public conceptualizes invasive species and responds to management strategies across diverse ecological and geographic contexts. However, only a limited number of studies have sought to simultaneously quantify the economic value that the public places on management, with most focusing on terrestrial species or a combination of terrestrial and aquatic contexts [1,2,9,32–34]. Studies that isolate preferences specific to aquatic systems are often limited to particular lakes [35], beaches/coasts [32], or state-managed water bodies such as parks and river systems [1], or are frequently focused on recreational users [9,36]. To our knowledge, McIntosh et al. [34] is the only study to date that quantifies the economic benefits of AIS management in freshwater systems from a broad public perspective. As a result, there exists limited empirical evidence on how the general public values AIS management beyond specific user groups and localized contexts.

Our study fills this gap by examining how the broader public values AIS management in a high-risk, freshwater-rich region—the state of Minnesota and its threatened $13.5 billion freshwater recreation economy [37,38]. This contrast between high economic stakes and proposed funding cuts raises critical questions about the sustainability of current AIS programs, and whether the level of investment reflects public support and priorities. While not all locations affected by AIS share Minnesota’s funding availability and management structure, growing pressures on state and national budgets and increasing reliance on local governments to shoulder program costs are not unique to the state. It is therefore critical to assess whether current and proposed investments in AIS management align with societal preferences and generate sufficient public benefits.

The primary objective of this paper is thus to estimate households’ willingness to pay (WTP) for AIS management, while simultaneously examining societal preferences related to these management practices. To this end, we conducted a survey of Minnesota residents within a contingent valuation (CV) framework, using a single-bounded, trichotomous-choice question format. Specifically, respondents were asked whether they would support an annual increase in their property tax or rent, with the resulting revenue earmarked for AIS management. Based on these responses, we estimate households’ WTP and examine whether and how it varies with behavioral tendencies, personal influences, socio-demographic characteristics, AIS-related knowledge, and individual avidity.

Our study draws on two distinct samples: (1) the general population of Minnesota and (2) a lake association sample within the state. The lake association sample was assembled through voluntary sign-ups on the website of Minnesota Lakes and Rivers Advocates (MLRA), a statewide advocacy group representing over 500 lake associations, mainly comprising people participating in lake-based community groups (further details on sample composition are provided in Section 2.1). In that sense, this sample is directly engaged with Minnesota’s aquatic ecosystems and may have a personal or financial interest in lake health and water quality. Accordingly, we hypothesize that lake association respondents, due to their ties to and direct interaction with water bodies, would exhibit higher levels of concern about AIS, perceive greater associated risks, and consequently place a higher value on AIS management compared to the general population.

Our study offers a state-level valuation in a high-risk context, defined by extensive freshwater resources and a growing presence of AIS. It is also, to our knowledge, the first to specifically target a distinct lake association sample, enabling a direct comparison between the general population and individuals with sustained and immediate ties to affected water bodies.

## 2 Methods

### 2.1 Survey

We conducted two self-administered surveys to assess public perceptions of AIS in Minnesota’s waters, targeting both general residents and lake association members (full copies of the survey will be provided upon request). The study protocol was reviewed and approved by the University of Minnesota’s Institutional Review Board (Study No. 00005269). All participants were adults. A cover letter containing consent information was sent to each potential participant, and completion and return of the survey indicated informed consent. For the general resident survey, we purchased a random sample of 2,000 individuals from Dynata, Inc., a commercial survey sampling provider. We adopt a modified version of the Tailored Design Method [39] and mailed out the survey packets with two subsequent follow-ups between May and September of 2021. Of the 2,000 mailings, 432 were returned as undeliverable, reducing the effective sample size to 1,568.

For the lake association survey, we collaborated with MLRA, a statewide nonprofit organization focused on lake stewardship. MLRA distributed the questionnaire via email to a curated list of 14,811 individuals who had previously signed up to receive communications. This list, developed and maintained over many years, primarily includes lake home and cabin owners, but also reaches some marina operators, anglers, and resort owners. The initial survey invitation was sent in August 2021, followed by two reminder emails through October 2021. Of those contacted, 3,046 recipients opened the email, and 32 messages bounced. While not a probability sample, the lake association list provides access to a population with strong ties to aquatic environments and a likely greater awareness of AIS-related issues. This group should therefore be interpreted as a self-selected stakeholder sample with elevated exposure to AIS, rather than as a representative sample of all nearby households.

Each survey contained 32 questions, with identical wording across the general and lake association versions to ensure comparability. The instrument was developed in consultation with AIS specialists at the Minnesota Department of Natural Resources (DNR), the Minnesota Aquatic Invasive Species Research Center (MAISRC), economists at the Minnesota Water Resources Center, and leaders of lake associations. Draft versions were informally pretested with colleagues and graduate students at the University of Minnesota to assess clarity. While we did not conduct formal focus groups or a standalone pilot study because of time and funding constraints, we refined the instrument through expert review and iterative revision.

**Fig 1:**
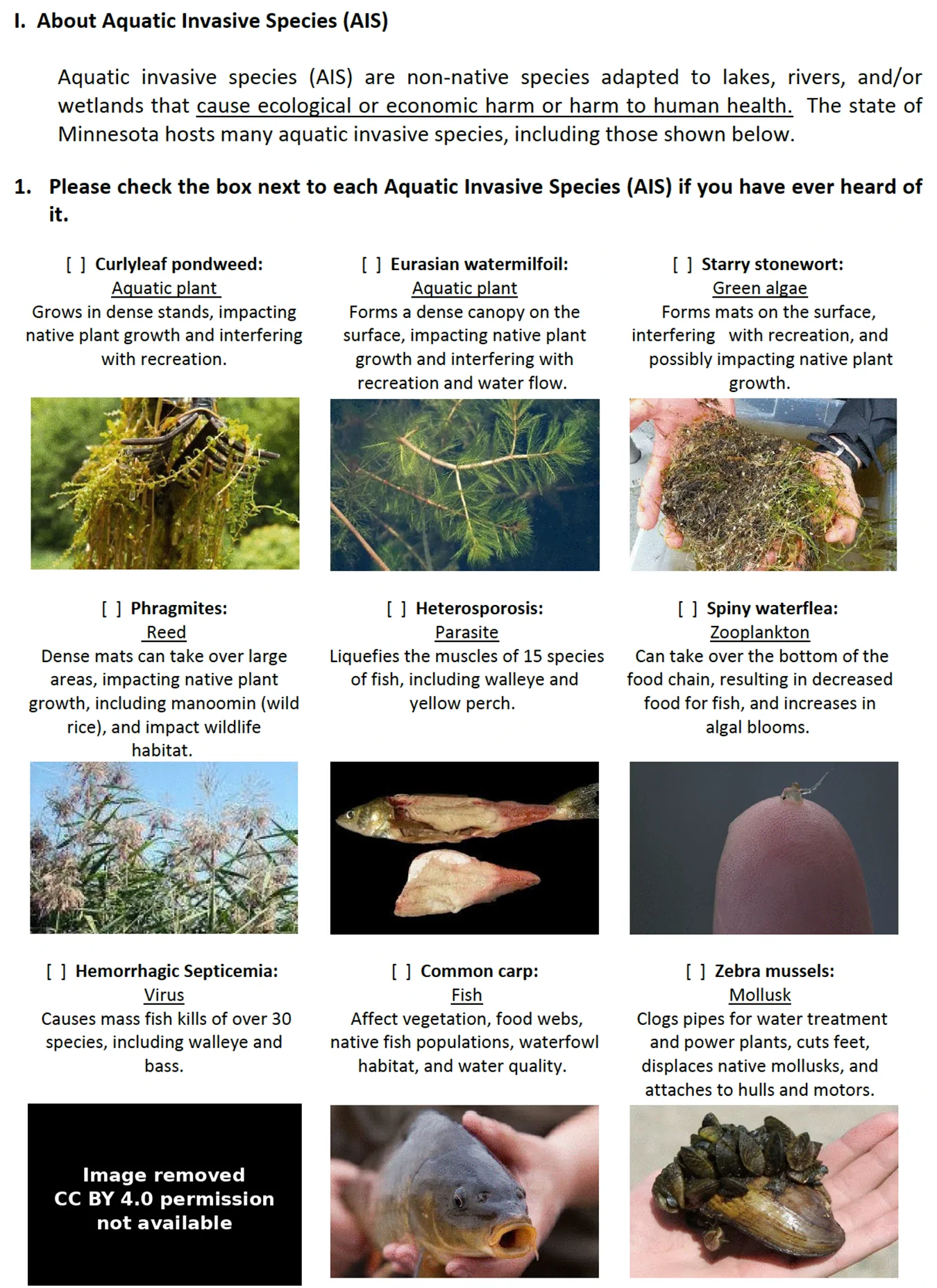
Aquatic invasive species shown in the survey. Images show curlyleaf pondweed, Eurasian watermilfoil, starry stonewort, Phragmites, Heterosporosis, spiny waterflea, common carp, and zebra mussels. The Hemorrhagic Septicemia image was removed because ownership and permission could not be confirmed. Reprinted from the Minnesota Aquatic Invasive Species Research Center (MAISRC), University of Minnesota, under a CC BY license, with permission from MAISRC.

The questionnaire began with a brief definition of AIS and an assessment of respondents’ familiarity with selected invasive species (see Fig 1). We focused on five species: common carp, zebra mussels, Eurasian watermilfoil, spiny waterflea, and starry stonewort. These species were selected because they are either well established or rapidly spreading in Minnesota, are consistently prioritized in state management and research programs, and together represent a range of ecological and economic impacts [40]. Additionally, we wanted to provide a variety of species to avoid the issue of survey takers transferring their love of a particular charismatic species [41], or rather hate of an uncharismatic species in this case, to the indicated program. To help respondents make informed judgments, we provided a brief introduction to each species at the beginning of the survey, including images, details about their habitats, and descriptions of their harmful impacts. This was followed by a series of questions gauging their knowledge, concerns, and attitudes toward AIS, as well as their connection to water bodies through lakeshore property ownership and/or recreational use. These plain-language descriptions and images, together with review of the instrument by AIS specialists at the Minnesota DNR, MAISRC, and the Minnesota Water Resources Center, were intended to support respondent comprehension before the elicitation.

**Fig 2:**
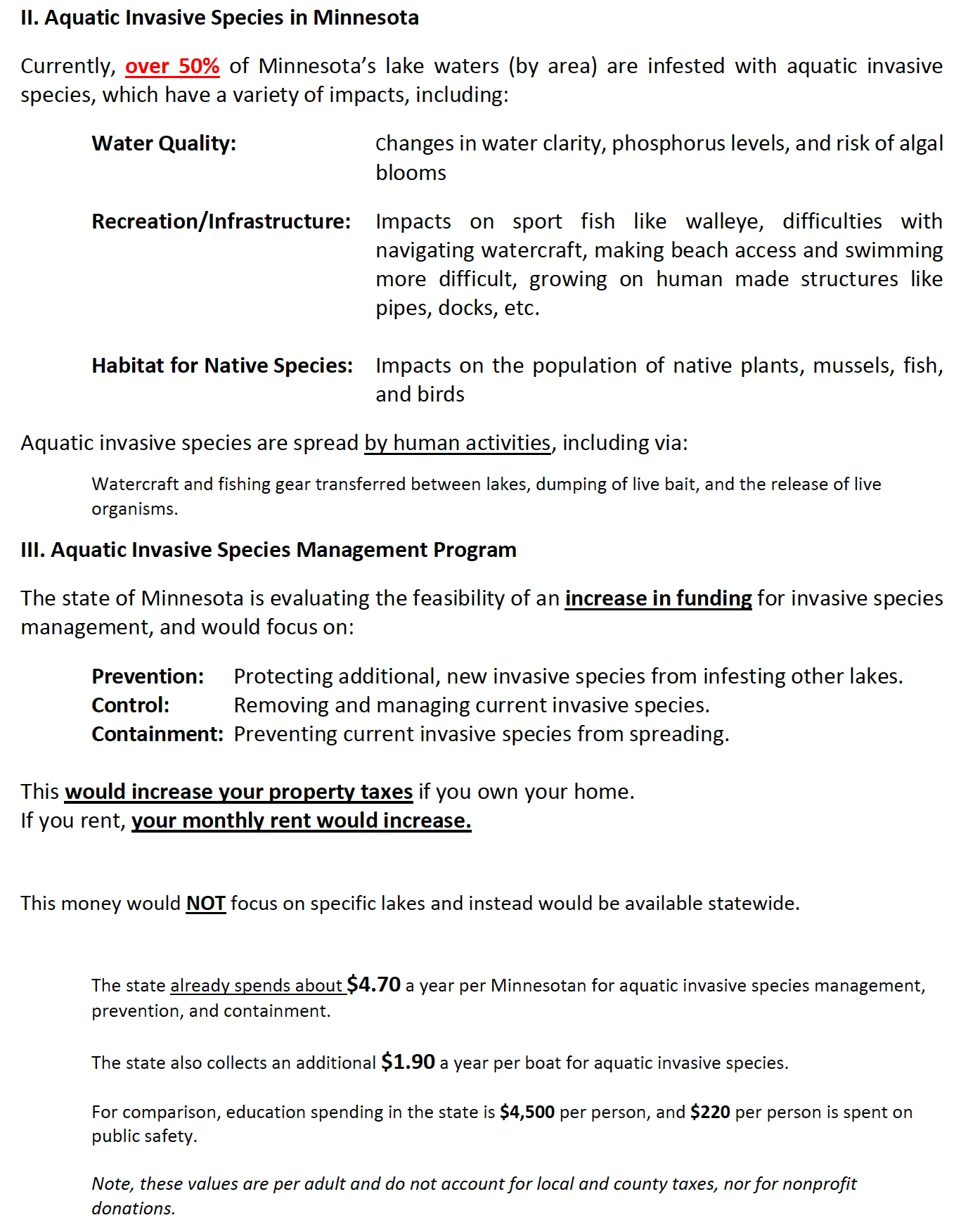
Aquatic invasive species (AIS) management scenario and proposed tax/rent increase presented to survey respondents.

We then provided an overview of the current state of AIS in Minnesota’s lakes, including major species and their ecological and recreational impacts. Next, we introduced the state of Minnesota’s consideration of increasing funding to implement comprehensive, state-wide AIS management strategies, including prevention, control, and containment efforts (see Fig 2). For context, respondents were reminded that the state already spends approximately $4.70 per person annually on AIS management and collects an additional $1.90 per boat as a dedicated surcharge on vessel registration. This surcharge was in place at the time of the survey and has since increased (See Minnesota Statutes §86B.415, Subd. 7: https://www.revisor.mn.gov/statutes/cite/86B.415).

The following variables were constructed from survey responses and are summarized in Table 2:

#### 2.1.1 Demographics.

Respondents provided demographic information including education and income. Education responses were re-categorized into a binary variable: respondents without a bachelor’s degree and those with at least a bachelor’s degree. Income data was collected by respondents choosing a pre-defined household income range.

#### 2.1.2 Risk perception.

**Fig 3:**
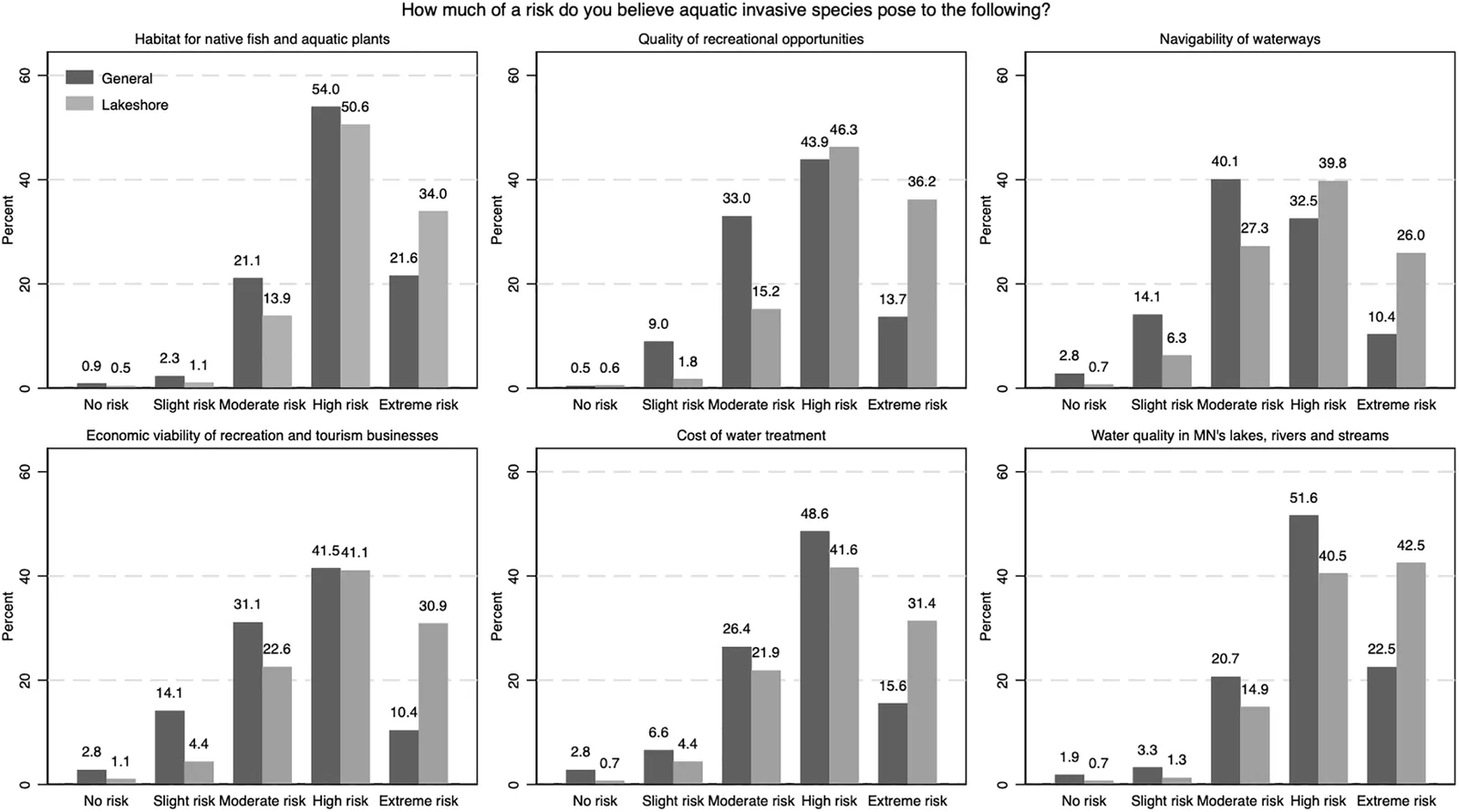
Perceived risk posed by aquatic invasive species to various water-related ecosystem services.

Respondents were asked to rate the risk posed by AIS on a 5-point Likert scale, from “no risk at all” to “extreme risk” for several water-related ecosystem services: “habitat for native fish and aquatic plants”, “quality of recreational opportunities (e.g., boating, fishing)”, “navigability of waterways”, “economic viability of recreation and tourism businesses”, “cost of water treatment”, and “water quality in Minnesota’s lakes, rivers, and streams”. We then averaged the responses to generate the risk perception variable (risk_percep). The nature and parameters of this variable are identical to those used by Levers and Pradhananga [9]; however, our method differs in construction: Levers and Pradhananga [9] summed the risk categories, whereas we used their mean. Fig 3 presents the full distribution of respondents’ perceived risk levels across each category for both general and lake association samples.

#### 2.1.3 Trust.

Respondents indicated the extent to which they trust various individuals or organizations for information about AIS on a five-point scale, ranging from “strongly distrust” to “strongly trust.” We provided a list of options, including “My friends and family,” “Department of Natural Resources,” “University Researchers/Extension,” “Environmental organizations,” “Bait shops/Sporting goods stores,” and “Fishing, boating, or sporting organizations.” In the lake association survey, two additional organizations were included—“Lake associations” and “Minnesota Lakes and Rivers Advocates”—given their more direct association with water bodies. We used principal component analysis (PCA) to reduce these trust items into two composite measures for the regression analysis. PCA is commonly used to summarize correlated variables into a smaller number of components when the objective is dimension reduction [42,43]. Given that these items have five ordered categories, they can generally be treated as approximately continuous for dimension reduction purposes [44], and we interpret the resulting scores as composite measures of trust for use in the regression models.

#### 2.1.4 AIS knowledge and concern.

Respondents were asked to rate their knowledge of AIS on a five-point scale, ranging from “not at all” to “extremely knowledgeable”, forming the AIS knowledge variable (AIS knowledge). They were also asked to rate their concern for each highlighted species on a five-point Likert scale, ranging from “not at all concerned” to “extremely concerned”, which were then averaged to generate the AIS concern variable (AIS concern).

#### 2.1.5 Value orientations.

To explore broader motivations behind public support for AIS management, we collected respondents’ views on environmental values, reflecting both ecocentric and anthropocentric perspectives. Specifically, we included statements representing instrumental and use-based orientations toward nature, such as “Nature’s value is to provide things useful to people” and “Fish are valuable only if people get to use them”, measured on a five-point Likert scale ranging from “strongly disagree” to “strongly agree”. While not included as regressors in our model, these orientations are drawn upon to contextualize and interpret patterns in the results.

#### 2.1.6 WTP elicitation.

We asked respondents about their willingness to financially support the management program described via an increase in property tax (or equivalent rent) and posed a single-bounded trichotomous-choice question—whether they would vote ‘for’, ‘against’, or ‘not sure’ if the revenue were earmarked solely for AIS management. Framing the choice as an increase in dedicated AIS funding mirrors the live legislative debate at the time of the survey over the level of state appropriation to the AIS Prevention Aid program.

We used a tax-based payment vehicle, consistent with previous CV studies [45–48]. A tax/rent instrument is appropriate for a non-excludable public good such as statewide AIS management, where a voluntary-payment vehicle would invite free-riding and exacerbate hypothetical bias [49]. This referendum-style framing aligns with established practice in CV studies, where it has frequently been applied to elicit support for funding environmental management programs rather than valuing precise ecological endpoints [2,29,33,35,50,51].

**Fig 4:**
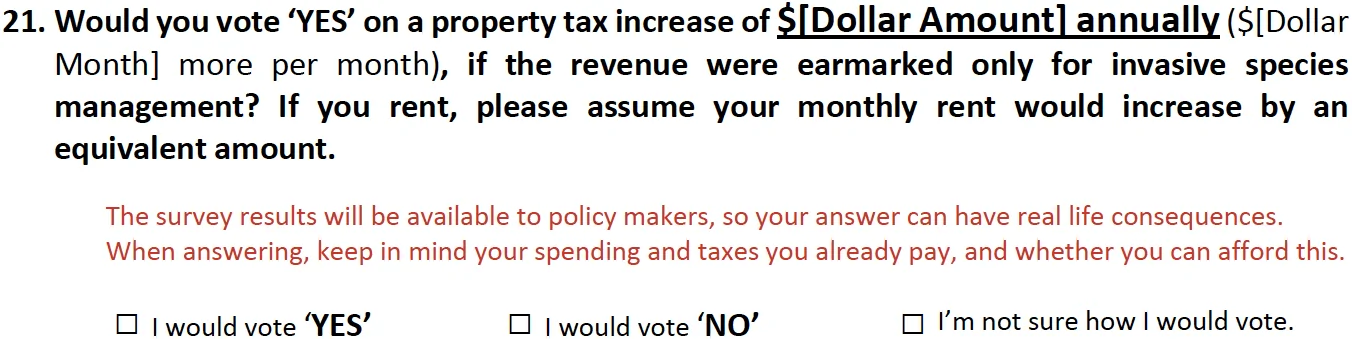
A trichotomous choice question asked to the respondents to elicit their willingness to pay.

The vehicle is also policy-realistic in Minnesota. Under Minn. Stat. 103B.501–581, Lake Improvement Districts levy property taxes specifically for AIS control and are established through county-board orders that may be initiated or contested by property-owner petition and referendum, giving both our payment vehicle and our referendum-style format direct real-world analogs. To reinforce the consequentiality of the vote, the elicitation question informed respondents that results would be shared with policymakers and asked them to weigh their existing taxes and ability to pay before voting (Fig 4); the trichotomous ‘for / against / not sure’ format also avoided forcing a directional response. The scenario itself described the program in terms of its goals (prevention, control, and containment) without attaching specific success rates, given the inherent uncertainty in projecting outcomes for a statewide multi-species program.

Fig 4 shows the exact wording of the question. Respondents were presented with a dollar amount (bid, hereafter) that mimicked a potential annual increase in property tax or rent, depending on their housing arrangement. These bid amounts, which took values $20, $40, $60, $80, or $100, were randomly and evenly assigned across the sample. Bid levels were determined based on professional judgment, informed by conversations with AIS experts and individuals from the Minnesota DNR. Respondents who voted ‘no’ or ‘not sure’ were further asked to elicit reasons for their decision. This helped determine whether their unwillingness or uncertainty was genuine or a form of protest against the program. We presented multiple predefined reasons, allowing respondents to check the appropriate boxes, while also giving them the flexibility to provide an open-ended response.

### 2.2 Empirical approach

We estimate the mean WTP, i.e., the welfare derived from the management of invasive species in Minnesota, using both a parametric and a non-parametric estimation framework.

The parametric approach requires that the percentage of respondents willing to vote for an additional tax should decrease as the amount of the tax, the bid amount, increases (monotonicity) [52]. In practice, survey data might exhibit non-monotonic patterns due to sampling variability, strategic behavior, or misunderstanding of the questions [53]. Additionally, ideal data would have no more than 20% of the ‘yes’ responses at the highest bid amount [54] and no more than 30% at any bid amount [55]. These thresholds are not optimization criteria but rather rule-of-thumb guidelines suggested in the CV literature to help ensure adequate variation across bid levels. In practice, however, achieving this might be challenging due to factors such as biases introduced by the survey design (e.g., anchoring), yea-saying behavior, respondents’ uncertainty (e.g., interpreting it as a broader environmental cause), or expressive voting intended to show support [54].

In CV studies, the former issue of non-monotonicity is referred to as the “flat-bid” curve, while the latter issue is known as the “fat-tail” problem. These response patterns complicate the estimation process and can lead to biased, inefficient, and/or unreliable estimates of WTP, particularly when parametric approaches like logit or probit models are used [53,56].

For all these reasons, CV practitioners often use a Turnbull estimator to estimate lower bound mean WTP [52,54,56–58]. The Turnbull estimator addresses issues of non-monotonicity, fat tails, and negative WTP in the following ways: first, it pools bid amounts and affirmative responses until the probability of a ‘yes’ response is either flat or decreases consistently as the bid increases; second, it addresses the fat-tail issue by excluding the upper tail of the bid distribution from the analysis; and third, it assumes a lower bound of zero for WTP, ensuring that estimates of expected WTP remain non-negative [56].

#### 2.2.1 *Parametric approach.*

Following the approach proposed by Cameron [59], we start with an assumption that the true WTP of respondent *i* is a linear function of the vector of explanatory variables, x, which could either be socio-demographic characteristics of the respondents, or their behavioral, personal, or attitudinal factors, or some combination thereof, such that:


$$\begin{array}{c} {WTP}_{i}=x_{i}^{\prime }\beta +\epsilon_{i},\;\epsilon_{i}\sim 𝒩(0,\sigma^{2}) \end{array}$$ (1)

where β is a vector of parameters associated with x and $\epsilon_{i}$ is the idiosyncratic error term, which is assumed to be distributed normally with mean 0 and variance $\sigma^{2}$. However, Eq. (1) is not directly estimable as we would not observe ${WTP}_{i}$; instead, we observe a randomly assigned bid value $B_{i}$ for each respondent and a trichotomous response to that bid, which takes the values ‘yes’, ‘no’, or ‘not sure’ (referred to as ‘don’t know’ or ‘DK’ hereinafter).

In this study, we follow a conservative approach and treat ‘DK’ as the ‘no’ response category—an approach consistent with the NOAA Panel’s recommendation that CV studies should adopt conservative estimation strategies [60]. Treating ‘DK’ as ‘no’ reduces the trichotomous response categories to dichotomous choice, ‘yes’ and ‘no’. Alternatively, one might eliminate ‘DK’ responses from the analysis. However, if the excluded samples differ significantly from those included in the analysis, this could lead to biased WTP estimates due to sample selection problems [61,62]. Further, it will decrease the efficiency of estimation through the reduction in sample size [63,64]. Another approach is to treat ‘DK’ as a middle response category between ‘yes’ and ‘no’ and model them using standard ordered probit or modified ordered probit model (also known as random valuation model), as in Wang [65], Alberini et al. [66], Champt et al. [33] and Shaikh et al. [67]. This method improves the model’s ability to handle ambiguous responses without discarding them or grouping them with ‘no’ responses, thereby reducing the risk of bias. However, its effectiveness is limited to “well-behaved” data—free of issues like non-monotonicity, “fat-tails” and “flat-bid” (see next subsection for details). These models, when applied *ex post* to our dataset, yielded unreliable and implausible estimates of WTP. So we excluded them from further analysis.

Given $x_{i}$, a respondent will accept a randomly assigned bid $B_{i}$ if their true WTP exceeds $B_{i}$. In this case, the probability function associated with the ‘yes’ response category is given by:


$$\begin{array}{cc} P(yes|B_{i},x_{i}) & =P({WTP}_{i}>B_{i})=P(x_{i}^{\prime }\beta +\epsilon_{i}>B_{i})=P\left(\frac{x_{i}^{\prime }\beta +\epsilon_{i}}{\sigma }>\frac{B_{i}}{\sigma }\right)\\ & =P\left(\frac{\epsilon_{i}}{\sigma }>\frac{-x_{i}^{\prime }\beta +B_{i}}{\sigma }\right)=1-P\left(\frac{\epsilon_{i}}{\sigma }\leq \frac{-x_{i}^{\prime }\beta +B_{i}}{\sigma }\right)\\ & =1-\Phi \left(\frac{-x_{i}^{\prime }\beta +B_{i}}{\sigma }\right) \end{array}$$ (2)

where Φ is the standard normal cumulative distribution function (CDF), and σ denotes the standard deviation of the error term. The complementary probability function for the ‘no’ response category will then be:


$$\begin{array}{cc} P(no|B_{i},x_{i}) & =1-P(yes|B_{i},x_{i})=\Phi \left(\frac{-x_{i}^{\prime }\beta +B_{i}}{\sigma }\right) \end{array}$$ (3)

Given Eqs. (2) and (3), we can write the log-likelihood function as:


$$\begin{array}{cc} \log L & =\sum_{i\in yes}\log\left[1-\Phi \left(\frac{-x_{i}^{\prime }\beta +B_{i}}{\sigma }\right)\right]+\sum_{i\in no}\log\left[\Phi \left(\frac{-x_{i}^{\prime }\beta +B_{i}}{\sigma }\right)\right] \end{array}$$ (4)

Our general estimation approach is to find the estimated parameters, $\hat{\beta }$, such that we can derive an estimated mean WTP as:


$$\begin{array}{c} E({WTP}_{i})=x_{i}^{\prime }\hat{\beta } \end{array}$$ (5)

#### 2.2.2 *Non-parametric approach: Turnbull estimates of the lower-bound mean.*

We use the same estimation approach as described in Haab and McConnell [52]. First, we calculate the total number of respondents at each bid level $B_{j}$, where j=1,...,M and *M* denotes the total number of distinct bid levels. Such total respondents $T_{j}$ is typically given by the summation of ‘yes’ $\left(Y_{j}\right)$ and ‘no’ $\left(N_{j}\right)$ responses, but can be the combination of all response categories. Then, we determine the proportion of ‘no’ responses at each bid level as $F_{j}=N_{j}/T_{j}$. This step requires us to code all other categories other than ‘yes’ to ‘no’. Thus, by design, this procedure yields conservative estimates of WTP.

If the proportion at the next bid level ($F_{j+1}$) is smaller than or equal to the current level ($F_{j}$), we pool adjacent bid levels and recalculate the proportion ($F_{j}^{*}$). We repeat this pooling process until all proportions are non-increasing. As such, there exists a bid level *M* + 1, where each respondent provides a ‘no’ response such that $F_{M+1}^{*}=1$. The probability density function $f_{j}^{*}$ is then given by $F_{j}^{*}-F_{j-1}^{*}$. Finally, we compute the lower bound estimate of WTP, $E_{LB}(WTP)$, using the following formula:


$$\begin{array}{c} E_{LB}(WTP)=\sum_{j=0}^{M^{*}}B_{j}\cdot f_{j+1}^{*} \end{array}$$ (6)

where $M^{*}$ is the number of new bid levels after pooling. To construct the confidence interval around this estimated lower bound of mean WTP, we compute the variance of $E_{LB}(WTP)$ using the following equation:


$$\begin{array}{c} V(E_{LB}(WTP))=\sum_{j=1}^{M^{*}}\frac{F_{j}^{*}(1-F_{j}^{*})}{T_{j}^{*}}\cdot {\left(B_{j}-B_{j-1}\right)}^{2} \end{array}$$ (7)

where $T_{j}^{*}$ represents the total number of observations at each pooled bid level.

## 3 Results

We gathered a total of 298 and 1,222 responses from the general resident and lake association surveys, respectively, corresponding to response rates of 19% and 8.3%. The response rate for the general sample was calculated using the effective sample size of 1,568. The response rate for the lake association sample was based on the number of delivered emails (14,779), defined as the total emails sent (14,811) minus bounced messages (32). These response rates are lower than those reported in many CV studies—such as Groothuis and Whitehead [63] (35%–41%), Cho et al. [68] (23%), Kobayashi et al. [69] (30%), McIntosh et al. [34] (35%), Chang et al. [70] (21%–28%), and Blaine and Lichtkoppler [71] (28.5%). This is consistent with the broad decline in survey response rates during the COVID-19 pandemic [72], and the lower rate for the lake association sample also reflects its email-based distribution, which typically yields lower response rates than mail surveys [73]. To investigate potential non-response bias, we performed a common proxy test by comparing early and late respondents, since late respondents are often considered more similar to non-respondents [71]. Except for visit frequency in the general resident sample and hiking activity in the lake association sample, we find no statistically significant differences between these groups, suggesting little to no evidence of systematic non-response bias.

In line with typical survey-based studies, our dataset encountered several cases of item non-responses for key variables of interest. Additionally, some respondents opted not to provide answers for certain socio-demographic characteristics, as permitted by the survey design. Consequently, we conducted a logical data cleaning process where we removed observations with missing data and “prefer not to respond” responses. This resulted in usable observations of 213 (13.6%) and 844 (5.7%) for the general and lake association samples, respectively. The non-response bias test was thus conducted using the final cleaned samples for each population. Early and late responders were identified based on the wave in which participants completed the survey. Respondents to the initial (first) wave of the mail and email distributions were identified as early responders, while those who responded to the second or third follow-up waves were categorized as late responders.

Table 1 presents the definition and description of key variables measured in our survey, while Table 2 presents their descriptive statistics. As expected by the survey design, the distribution of bids across the general and lake association samples is similar. While both samples are predominantly male, they differ in age distribution: approximately 95% of lake association respondents are 45 years or older, compared to about 75% in the general sample. We find that 61% of general residents have attained at least a bachelor’s degree, compared to 77% of lake association members. The average income level in both the lake association and general resident samples falls within the “$75,000–$99,999” range.

**Table 1:** Description and definition of the variables.

Variables	Definition	Description
bid	Dollar amount presented to the respondents as an increase in property tax/rent	Takes values of $20, $40, $60, $80, and $100
age	Age of the respondents	1 if age ≥ 45
female	Gender of the respondents	1 if female
educ	Highest level of formal education	1 if at least have a bachelor’s degree
income	Household income in USD	1 = under $20,000; 2 = $20,000 to $49,999; 3 = $50,000 to $74,999; 4 = $75,000 to $99,999; 5 = $100,000 to $149,999; 6 = $150,000 to $199,999; 7 = $200,000 or more
visit freq	Frequency of visit to lake, river, stream, or creek in the last 12 months	1 = did not visit; 2 = once; 3 = 2–5 times; 4 = 6–11 times; 5 = 12 times or more
risk percep	Perception of risk towards AIS constructed based on the extent to which respondents believe AIS poses a risk to various water-related ecosystem services, such as “habitat for native fish and aquatic plants”, “quality of recreational opportunities (e.g., boating, fishing)”, “navigability of water-ways”, “economic viability of recreation and tourism businesses”, “cost of water treatment”, and “water quality in Minnesota’s lakes, rivers, and streams”.	1 = no risk at all; 2 = slight risk; 3 = moderate risk; 4 = high risk; 5 = extreme risk
trustscore1	Composite score generated from principal component analysis using the trust rank of categories “Department of Natural Resources”, “University Researchers/Extension”, and “Environmental organizations”. Two additional categories, “Lake associations” and “Minnesota Lakes and Rivers advocates”, were included for the lakeshore survey.	1 = strongly distrust; 2 = somewhat distrust; 3 = neither trust nor distrust; 4 = somewhat trust; 5 = strongly trust
trustscore2	Composite score generated from principal component analysis using the trust rank of categories “Bait shops/Sporting goods stores” and “Fishing, boating, or sporting organizations”.	1 = strongly distrust; 2 = somewhat distrust; 3 = neither trust nor distrust; 4 = somewhat trust; 5 = strongly trust
fishing	Respondents’ participation in fishing as an activity	1 if fishing
hiking	Respondents’ participation in hiking as an activity	1 if hiking
motorboating	Respondents’ participation in motorboating as an activity	1 if motorboating
AIS knowledge	Respondents’ knowledge about AIS	1 = not at all; 2 = slightly; 3 = moderately; 4 = very; 5 = extremely
AIS concern	Respondents’ concern towards AIS generated based on five species: Common carp, Zebra mussels, Eurasian watermilfoil, Spiny waterflea, and Starry stonewort.	1 = not at all concerned; 2 = slightly concerned; 3 = moderately concerned; 4 = very concerned; 5 = extremely concerned
lakeshore	Ownership of lakeshore property	1 if own a lakeshore property

Note: Age and education were recategorized into binary variables: under 45 years versus 45 years or older, and without a bachelor’s degree versus at least a bachelor’s degree. Household income was recategorized to align with American Community Survey 2020 groupings, with $200,000 to $249,999, $250,000 to $299,999, and $300,000 or more collapsed into a single “$200,000+” category.

**Table 2:** Descriptive statistics of the variables used in the analysis.

Variables	General sample			Lakeshore sample		
	Mean	Std. dev.	Min; max	Mean	Std. dev.	Min; max
bid	59.906	29.936	20; 100	60.000	28.668	20; 100
age	0.742	0.439	0; 1	0.956	0.205	0; 1
female	0.455	0.499	0; 1	0.326	0.469	0; 1
education	0.610	0.489	0; 1	0.770	0.421	0; 1
income	4.056	1.715	1; 7	4.927	1.517	1; 7
visit freq	3.638	1.275	1; 5	3.995	1.377	1; 5
risk percep	3.646	0.719	1; 5	4.057	0.674	1; 5
trustscore1	0.061	1.512	−5.707; 1.680	0.146	1.636	−7.771; 2.610
trustscore2	−0.038	1.374	−3.280; 2.761	−0.027	1.311	−3.632; 3.660
fishing	0.400	0.491	0; 1	0.473	0.500	0; 1
hiking	0.530	0.501	0; 1	0.432	0.495	0; 1
motorboating	0.282	0.451	0; 1	0.495	0.500	0; 1
AIS knowledge	2.385	0.759	1; 5	3.089	0.777	1; 5
AIS concern	3.298	0.892	1; 5	3.849	0.712	1; 5
lakeshore	0.235	0.425	0; 1	1.000	0.000	1; 1
N	213			844		

Note: Std. dev. = standard deviation, min = minimum, max = maximum, and N = number of observations.

To evaluate the representativeness of our data, we compare the demographic characteristics of the general resident sample with those of the Minnesota population as reported in the American Community Survey (2020) by conducting chi-square tests across age, education, income, and gender (S1 Table in the Supporting Information). We find that the general sample differs significantly from the Minnesota population in age, education, and income, but not in gender. Accordingly, the general sample was weighted using iterative proportional fitting (also known as raking) on age, education, and income to align with ACS benchmarks; the weighting was implemented using the *ipfweight* command in Stata. S1 Table also presents the comparison of the lake association sample. Although this group differs significantly from the Minnesota population across all demographic characteristics, it was not weighted because it is not intended to represent the statewide population.

As discussed in the survey section, we generated variables regarding Risk Perception, AIS Knowledge, AIS Concern, Visit Frequency and Type, and Trust.

On average, both general and lake association respondents visited water bodies around 6–10 times annually, with mean values of 3.6 and 4.0, respectively. A large proportion of respondents participated in activities such as fishing, hiking, and motorboating. As expected, respondents in the lake association sample showed higher engagement in fishing and motorboating due to their direct access to water bodies—100% of them own lakeshore property compared to only 23.5% of general residents (see lakeshore in Table 2)—while hiking was relatively more popular among the general residents.

We find a mean risk perception score, risk_percep, of 3.6 for respondents in the general sample and 4.1 for those in the lake association sample, (statistically significant at 1% level with t-stat = 7.846, p-value = 0.000). This indicates that while general residents perceive AIS as posing a moderate to high risk, lake association respondents view them as a high to extreme risk. This result is consistent across all six domains of water-related ecosystem services (see Fig 3). For example, 84.4% of lake association respondents consider AIS a high to extreme risk to native fish and aquatic plant habitats, compared to 75.6% of general respondents. A similar trend is evident in perceptions of risk to water treatment costs and the economic viability of recreation and tourism. The AIS knowledge variable also shows higher levels for lake association respondents, where they report being moderately to very knowledgeable (mean = 3.1) compared to general residents, who report being slightly to moderately knowledgeable (mean = 2.4), statistically significant difference at 1% level (t-stat = 11.872, p-value = 0.000). Lake association respondents also express somewhat greater concern about AIS (statistically significant at 1% level with t-stat = 9.546, p-value = 0.000), as indicated by a mean concern score of 3.9 compared to 3.3 among general residents (see AIS concern variable).

For the trust component, we first excluded the “My friends and family” category, as this source is often subjectively biased. PCA of the remaining trust items, retaining components with eigenvalues greater than one, produced two components. The first component groups trust in the “Department of Natural Resources,” “University Researchers/Extension,” and “Environmental organizations,” and is reflected in trustscore1. For the lake association survey, this component also includes trust in “Lake associations” and “Minnesota Lakes and Rivers Advocates.” The second component groups trust in “Bait shops/sporting goods stores” and “Fishing, boating, or sporting organizations,” and is reflected in trustscore2.

The PCA diagnostics support this two-component solution. Bartlett’s test of sphericity was rejected at 1% significance level in both samples, indicating that the trust items are sufficiently intercorrelated for PCA. The KMO measure of sampling adequacy was 0.61 for the general sample and 0.69 for the lake association sample, both exceeding the conventional 0.50 minimum [74,75]. Component 1 showed acceptable to good internal consistency (Cronbach’s α = 0.84 and 0.79); because Component 2 comprises only two items, for which alpha is not an informative reliability measure, we report its inter-item correlation instead (*r* = 0.81 and 0.67). The two components together explain 82.7% of the variance in the general sample and 65.0% in the lake association sample. Full diagnostics, rotated component coefficients, and sample sizes are reported in S2 Table.

We find a slightly higher trustscore1 (0.15) for the lake association respondents compared to general residents (0.06), indicating that both groups generally trust the information sources, but with only a marginal difference. In contrast, we find negative trustscore2 for both general and lake association respondents (mean score of −0.04 and −0.03), which indicates that respondents either show less trust or are more critical of these businesses and organizations.

A formal analysis of value orientations using the same general resident dataset analyzed in this study is presented in Pradhananga et al. [31], where the authors find that anthropocentric values are associated with lower perceived risk and reduced confidence in the effectiveness of AIS-related actions, whereas ecocentric values are positively associated with response efficacy. In this paper, we do not model those relationships; instead, we report descriptive statistics for both the general resident and lake association samples to enable comparison with lake association members, a group not included in the prior analysis (see S3 Table). This comparison allows us to examine whether environmental values differ across populations with varying exposure to AIS. While they may not directly influence WTP, these values help interpret why residents may support public investment in AIS management even in cases where personal benefits are not immediate or direct.

We find that ecocentric views were common in both samples. Over 60% of general residents and lake association members agreed that protecting the environment is more important than providing fishing opportunities, and more than half affirmed that fish have intrinsic rights to exist. In contrast, fewer than 10% in either group agreed with statements emphasizing the instrumental or economic utility of fish or nature. Lake association residents showed slightly stronger ecocentric leanings overall—for example, they expressed lower agreement with the view that “fish are primarily valuable as food for people”.

### 3.1 WTP estimation

Table 3 presents a distribution of responses to the WTP question across both surveys. Within the range of potential donations offered in the survey ($20–$100 per household per year), 54% of general residents and 80% of lake association members voted ‘yes’ to support state-wide AIS management, reflecting support conditional on the specified tax/rent increases.

**Table 3:** Distribution of responses in % to the WTP question.

Response	General sample	Lakeshore sample
yes	53.52	79.74
no	7.98	3.55
protest No	16.43	4.27
DK	22.06	12.55
N	213	844

Note: DK = don’t know, N = number of observations, and WTP = willingness to pay.

The general residents recorded a higher proportion of protest responses compared to the lake association members (16.43% vs. 4.27%, see Table 3). The proportion of protest responses in the general resident survey is lower than the 23% reported in Levers and Pradhananga [9], which also studies AIS, but focuses on boaters who already pay for AIS management as part of their boating registrations and out of state fishing licenses. The proportion does align with other environmental studies such as Strazzera et al. [76] (17.75%), Martin-Lopez et al. [77] (18.6%), Lyssenko and Martinez-Espineira [78] (16.3%), and the meta-study on protest responses by Meyerhoff and Liebe [79] (17.69%) (see Table 4).

**Table 4:** Distribution in % of reasoning associated with the “protest No” category.

Protest reasons	General sample	Lakeshore sample
I would not support an increase in property taxes regardless of the amount or what it is for	54.29	25.00
I would support taxes for individual lakes, but not for the state as a whole	11.43	11.11
Distrust on government and/or program	17.14	33.33
Finance through other means and taxes or reallocation	5.71	27.78
Others or everyone should pay	11.43	2.78
N	35	36

Note: N = number of observations.

These protest responses raise important concerns regarding how they should be treated during estimation. The conventional strategy is to either treat protest responses as ‘no’ or exclude them from the sample, similar to how ‘DK’ responses would be handled. However, treating them as ‘no’ may lead to underestimation of WTP, while exclusion can result in biased estimates of WTP, particularly if the excluded sample differs significantly from the sample used in analysis [68,80,81]. Researchers often use the Heckman sample selection model to address selectivity bias; however, this approach comes at the cost of reduced efficiency due to a reduced sample size [62]. Alternatively, one can treat protest responses as an intermediate category between positive and zero bids, as in Cho et al. [68], and measure the welfare using an ordered probit model. However, in the presence of ‘DK’ responses, the ordering of the protest and ‘DK’ categories becomes less clear. There is neither a consensus in the literature nor statistical guidance on how to model these categories.

Given our modest sample size, we follow the conventional strategy of treating protest responses as ‘no’ responses, which reduces the response categories to a dichotomous choice between ‘yes’ and ‘no’, as we also coded ‘DK’ responses as ‘no’. This approach yields conservative estimates of mean WTP.

Table 5 presents the estimates from the maximum likelihood estimation for the general resident survey, along with the estimated mean WTP and its confidence interval. The first three columns of Table 5 present the results for the unweighted sample, while the last three columns report the corresponding analysis for the weighted sample, adjusted to match the Minnesota population distribution of age, education, and income. Alternatively, one could run a standard probit model and use those estimates to derive results equivalent to those obtained via maximum likelihood estimation. We report the results from the probit model in S4 Table.

**Table 5:** Estimates from maximum likelihood estimation for the general resident survey.

Variables	Unweighted			Weighted		
	(1)	(2)	(3)	(4)	(5)	(6)
age	−35.583	–	–	12.758	–	–
	(0.810)	–	–	(0.300)	–	–
female	−33.121	–	–	−10.985	–	–
	(0.760)	–	–	(0.300)	–	–
educ	−0.820	–	–	10.068	–	–
	(0.020)	–	–	(0.240)	–	–
income	11.045	–	–	2.954	–	–
	(0.800)	–	–	(0.270)	–	–
visit freq	20.594	–	–	20.965	–	–
	(1.040)	–	–	(1.070)	–	–
risk percep	46.809	47.053	–	93.481	78.682*	–
	(1.060)	(1.460)	–	(1.590)	(1.810)	–
trustscore1	44.432	39.969*	–	37.227	39.977*	–
	(1.500)	(1.740)	–	(1.540)	(1.700)	–
trustscore2	−16.104	−18.837	–	−18.056	−24.660	–
	(0.960)	(1.320)	–	(1.050)	(1.450)	–
fishing	−39.773	–	–	−60.612	–	–
	(0.860)	–	–	(1.240)	–	–
hiking	48.325	53.864	–	58.402	71.338	–
	(1.060)	(1.370)	–	(1.260)	(1.540)	–
motorboating	49.558	49.764	–	105.736*	90.764*	–
	(1.080)	(1.300)	–	(1.800)	(1.850)	–
AIS concern	27.370	–	–	1.306	–	–
	(0.830)	–	–	(0.050)	–	–
AIS knowledge	−19.109	–	–	−10.594	–	–
	(0.650)	–	–	(0.430)	–	–
lakeshore	−26.586	–	–	−24.488	–	–
	(0.550)	–	–	(0.510)	–	–
constant	−201.449	−142.876	75.614***	−389.620	−284.840	57.396**
	(0.970)	(1.100)	(4.420)	(1.420)	(1.560)	(2.420)
N	213	213	213	213	213	213
%yes	53.521	53.521	53.521	53.521	53.521	53.521
sigma	179.036*	161.329**	174.434**	145.664*	140.045**	219.324**
	(103.153)	(79.946)	(88.636)	(77.995)	(70.319)	(169.614)
Log Lik.	−124.938	−128.448	−145.150	−114.056	−116.461	−146.370
AIC	281.876	270.896	294.301	260.112	246.921	296.740
BIC	335.656	294.425	301.023	313.892	270.451	303.462
WTP	76.854***	74.168***	75.614***	75.670***	68.477***	57.396**
	(19.361)	(16.756)	(17.104)	(18.137)	(16.155)	(23.685)
C.I.	[-14.81; 213.52]	[31.13; 182.25]	[22.03; 183.39]	[7.01; 188.95]	[17.59; 159.89]	[-108.35; 215.80]

Note: WTP = willingness to pay in USD per household per year. AIC = Akaike information criterion. BIC = Bayesian information criterion. σ = sigma. C.I. = confidence interval. N = number of observations. Robust standard errors are provided in parentheses. * *p* < 0.10, ** *p* < 0.05, *** *p* < 0.01.

We find statistically significant estimates of mean WTP across all maximum likelihood specifications, ranging from $57.40 to $76.85 per household per year. The unweighted model with a full set of variables (Table 5, column (1)) generates the highest WTP estimate, $76.85, yet none of the included variables has a statistically significant effect on WTP. To examine whether multicollinearity may contribute to this result, we assess correlations among the covariates following Greene [62]. The Spearman rank correlation matrix (S5 Table) shows that the highest correlation is between AIS concern and risk percep (0.666). This is expected, as respondents who are more concerned about AIS are likely to perceive it as posing higher risk. The remaining statistically significant correlations, including that between education and income, are relatively weak and do not appear to pose a serious multicollinearity issue overall.

The weighted counterpart of the full model (column (4)) yields a mean WTP estimate nearly identical to the unweighted full model ($75.67 vs. $76.85), although weighting modestly changes individual coefficient estimates and their statistical significance. In the weighted full model, motorboating becomes statistically significant at the 10% level, while the remaining covariates remain insignificant. Because the weights adjust the relative influence of observations across age, education, and income groups, these shifts indicate that individual coefficient estimates are somewhat sensitive to the demographic composition of the sample, even when the implied mean WTP is not. Model fit improves under weighting (lower AIC/BIC), but because most coefficients remain imprecisely estimated, we do not interpret their magnitudes.

Next, we vary the choice of explanatory variables and repeat our analysis. Specifically, we include risk percep, trustscore1, trustscore2, hiking, and motorboating in the analysis. We report the effects of these variables in column (2) of Table 5. These variables again show no significant effect on WTP, except trustscore1 at the 10% level. Despite this, the estimated WTP remains highly significant at the 1% level. In the weighted reduced model (column (5)), trustscore1 remains weakly significant at the 10% level, and risk percep and motorboating are also significant at the 10% level. The estimated mean WTP declines modestly from $74.17 in column (2) to $68.48 in column (5). The probit specification yields qualitatively similar results, with income, risk_percep, trustscore1, trustscore2, hiking, and motorboating being significant at the 1% to 5% levels (S4 Table).

Finally, we estimate WTP by excluding all explanatory variables from the model (i.e., a constant-only model; see column (3) of Table 5) and find a WTP estimate of $75.61 per household per year. This is close to the estimates from the full and reduced specifications ($76.85 and $74.17, respectively), indicating that within the unweighted sample the mean WTP is not sensitive to the choice of covariates. In the weighted constant-only model (column (6)), the mean WTP is $57.40, which is lower than the unweighted counterpart. This decline reflects the application of population weights: groups with relatively higher stated WTP—specifically older, more educated, and higher-income respondents—are overrepresented in the unweighted sample, and raking reduces their influence. Without covariates to absorb these compositional differences, the constant-only specification shows the largest weighting effect.

We repeated the analysis after removing both the protest and ‘DK’ response categories. This reduced the sample from 213 to 131 observations and raised the share of “yes” responses from 54% to 87%. With only 17 “no” responses remaining out of 131, the data did not contain enough variation in the dependent variable to identify the parameters of the maximum likelihood model, and none of the three parametric specifications (full, reduced, or constant-only) converged. The non-parametric Turnbull estimator does not require parameter identification and so remained defined, with the lower-bound mean rising from $34.66 to $70.34. This increase is consistent with the underlying selection effect, since protest and DK respondents are systematically less willing to pay and excluding them inflates the estimated WTP. We therefore do not treat these results as an alternative to our primary specification, as the resulting subsample is no longer representative of the general resident population.

We report the 95% confidence interval associated with these mean WTP estimates in the last row of Table 5. These intervals are calculated using the Krinsky-Robb method based on 10,000 draws [82], as suggested in Haab and McConnell [52]. Across all specifications, the confidence intervals are wide and often exceed the bid range. Some models, i.e., Table 5, column (1) and (6), yield negative lower bounds, which undermine the reliability of the mean WTP estimates for inference. Large confidence intervals often arise from low-bid levels, where the probability of a ‘yes’ response is insensitive to changes in bid amounts [83]. Literature recognizes this issue as a “fat-tail” phenomenon, characterized by a high percentage of respondents answering ‘yes’ even at the highest bid levels.

We explore the distribution of ‘yes’ responses at each bid level in both samples (general and lake association) and present them in Table 6. Two key observations emerge: first, the *implicit inverse demand curve* for the lake association sample, as would be given by the plot of the percentage of ‘yes’ responses against bid amounts, appears almost flat; second, both general and lake association samples experience the “fat-tail” issue, with more than 50% of respondents saying ‘yes’ at the highest bid ($100). This pattern suggests that our upper bid was insufficient to capture the upper tail of the WTP distribution, particularly for the lake association sample. While we analyze the lake association sample using the same parametric framework as for the general resident sample, we do not report those results as they yield highly inflated, unreliable, and implausible estimates of WTP with exceedingly large confidence intervals. This issue primarily arises from the low quality of the lakeshore dataset for parametric analysis, as it is affected by both flat bid and fat-tail phenomena, as discussed above and illustrated in Table 6. This problem worsens when ‘DK’ and ‘protest’ response categories are removed from the sample (see S6 Table).

**Table 6:** Percentage of yes at each bid level.

	General sample		Lakeshore sample	
Bid	% yes	n	% yes	n
20	64.58	48	83.72	172
40	62.79	43	80.00	175
60	45.71	35	79.74	153
80	36.11	36	77.51	169
100	52.94	51	77.71	175

Note: n = number of observations at each bid level.

Accordingly, we apply the Turnbull method to analyze the general and lake association sample, with the empirical framework presented in Section 2.2. Table 7 presents the lower bound mean WTP estimates using the Turnbull estimation approach, along with their 95% confidence intervals for both the reduced and full samples. The reduced sample includes the same set of observations as we use (or would use) for general residents (for the lake association sample), while the full sample includes all observations prior to data cleaning. Since the Turnbull method provides parameter-free estimates of WTP, we were able to use the full sample, as it requires only the bid and response variables—both of which contained no missing data.

**Table 7:** Turnbull estimates of mean lower bound.

	Reduced sample		Full sample	
	General	Lakeshore	General	Lakeshore
mean	34.655	64.215	39.660	74.550
95% C.I.	[30.319; 38.990]	[61.974; 66.456]	[34.792; 44.528]	[72.111; 76.989]
N	213	844	291	1222

Note: N = number of observations, and C.I. = confidence interval. Estimates are in USD per household per year.

As can be seen in Table 7, there are significant differences in the lower bound mean WTP estimates between the general and lake association respondents. In the reduced sample, the mean WTP associated with general residents is $34.66 per household per year, which is substantially lower than the estimate of $64.22 for lake association respondents. While the estimates of WTP for lake association members also remain higher in the full sample analysis, these estimates increase for both groups (to $39.66 and $74.55 for general and lake association residents, respectively). Our estimates of WTP, particularly the $35 to $40 range for general residents, though conservative, are consistent with the findings of McIntosh et al. [34], who report household WTP values between $34 and $48 as a one-time donation to delay the onset of low- to high-impact AIS scenarios in inland U.S. water bodies.

## 4 Discussion

Understanding the public’s viewpoint regarding conservation goals is vital as policymakers and public resource managers must balance finite resources against competing needs. The goal of managing invasive species, one of the top drivers of extinction, is no different.

This AIS study uniquely samples respondents from both a statewide general population and an advocacy group, which helps show how residents as a whole think, as well as those from community organizations. The advocacy group studied here, from MLRA, is not a group dedicated solely to invasive species, nor specifically environmental causes. In fact, the organization began as and continues to be an advocate for reduced property taxes and its members are evenly divided between political parties [84]. Nonetheless, many of its respondents as well as those from the general sample are pro tax-based AIS management with 54% of general residents and 80% of lake association members voting ‘yes’ to support a state-wide property tax increase earmarked for AIS. Perhaps surprisingly, given the anti-tax history of MLRA, the lake association respondents are willing to pay more than general residents for statewide AIS management, prevention, and control, with Turnbull lower-bound estimates of $64 to $75 vs $35 to $40 annually, respectively. These estimates should be interpreted as conservative lower bounds rather than precise valuations, given the wide confidence intervals in the parametric models and the flat-bid and fat-tail features of the data. Future research in similar high-engagement contexts should extend the bid range upward to better capture the upper tail of the WTP distribution, test alternative payment vehicles, and consider stratified sampling across additional stakeholder groups (for example, anglers and boaters) to support more structured comparison than the two-sample contrast we present here.

Given that the lake association sample is a self-selected stakeholder group with direct ties to affected water bodies, their strong support is logical. This is mirrored by our finding that people who engage in boating report higher WTP for AIS management, a pattern consistent with the direct benefit they would receive given that aquatic invasive plants, such as Eurasian watermilfoil and starry stonewort, impede the movement of boats.

The difference in WTP between the lake association and general samples may reflect differences in underlying environmental value orientations. As discussed in Section 2.1 and shown in S3 Table, both groups expressed strong ecocentric leanings, but lake association respondents were even less likely to agree with instrumental statements such as “Fish are valuable only if people get to use them.” They also show higher AIS perceived risk, knowledge, and concern than that of the general population. Sorting may be a factor as individuals with stronger preferences for lake-related amenities may be more likely to select into a lake association or have developed stronger preferences during their time as a member of a lake association.

While all of the respondents of the lake association survey are property owners, owning lakeshore property does not automatically make one a lake association member. As such, simply owning lakefront property may not be the driving force behind the increase in WTP, evidenced by our showing no increase in WTP for general residents who happen to also own lakeshore property. We also find no impact on support from AIS knowledge, a result found in previous studies as well [85]. Respondents do show fairly high AIS knowledge, which may be high in Minnesota due to the emphasis on lake culture. Expanding future surveys to incorporate explicit ecological baselines and probabilities of program success could require a high respondent learning curve–an important caveat.

The higher protest rate among general residents may reflect opposition to property tax increases and distrust of state-level institutions, the two most commonly cited reasons in Table 4. Smaller shares cited preferences for funding individual lakes rather than statewide programs, or the view that others should bear the cost. The presence of distrust as a stated reason is consistent with prior work finding that beliefs about program ineffectiveness are associated with lower support for invasive species management [86].

Trust in entities that perform AIS management is an important factor associated with WTP. Individuals who report higher trust in the DNR, the University, and environmental organizations also report higher WTP, with the coefficient on trustscore1 positive across all parametric specifications and significant at the 1% level in the probit model (S4 Table). This pattern is consistent with prior research [86,87] and with the protest-response reasonings in Table 4, where distrust of government and program effectiveness was a leading reason cited for opposition among general residents.

Our findings underscore substantial public support for managing AIS, reflecting widespread recognition of their ecological and economic threats. Residents, even those who do not have strong direct connections to aquatic ecosystems, find value in AIS management. In light of recent changes to AIS management funding in Minnesota, and ongoing debate, these results suggest that continued or increased state investment would be broadly consistent with public preferences within Minnesota. We caution against extending these point estimates to other states or contexts: the magnitude of WTP is likely to depend on local ecological conditions, the extent of existing infestation, recreational use patterns, and cultural ties to freshwater systems, all of which are particularly pronounced in Minnesota. Our findings may be most informative for jurisdictions with comparable freshwater resources and stakeholder structures. Support for this conservation program may be shaped by direct exposure, self-selection, and trust in managing organizations, ergo support should not be attributed solely to proximity. Nevertheless, the differences in WTP between the lake association and general samples warrant attention from policymakers when considering support for local organizations and opportunities for more targeted cost-sharing and outreach strategies.

A key message from the Intergovernmental Science-Policy Platform on Biodiversity and Ecosystem Services’s assessment report on invasive species is that engagement and collaboration with stakeholders, Indigenous Peoples, and local communities improves invasive species management outcomes [88]. Our findings reinforce this view. Respondents who reported higher trust in the DNR, the University, and environmental organizations also reported higher WTP, and protest responses among general residents often pointed to distrust of government and program effectiveness. These results suggest that securing public investment in AIS management is not only a technical or budgetary problem but also a trust-building problem. Fostering trust between natural resource managers, community organizations, and the general public may have the largest potential impact on program support, and our results suggest that investments in public outreach and trust-building may be an important complement to the technical management of AIS itself.

## Supporting information

S1 Table Sample and population distribution (in %) of demographic characteristics of MN. (PDF)

S2 Table Principal component analysis diagnostics and rotated component coefficients. (PDF)

S3 Table Respondents’ agreement with statements reflecting ecocentric and anthropocentric values. (PDF)

S4 Table Estimates from the probit model for the general resident survey. (PDF)

S5 Table Spearman’s rank correlation matrix for general resident survey. (PDF)

S6 Table Percentage of yes and total sample in each bid amount after eliminating protest and ‘DK’ categories from the sample. (PDF)
