# Supplementary material for: Economic benefits of aquatic invasive species management in freshwater systems: A contingent valuation study
Source: PLoS One. 2026 Sep 16;21(9):e0346515. doi: 10.1371/journal.pone.0346515 (PMC13581201; doi:10.1371/journal.pone.0346515)
Supplement: Supplementary file 1 [file pone.0346515.s001.pdf]

S1 Table. Sample and population distribution (in %) of demographic characteristics of MN.

Characteristic	Category	General sample	Lakeshore sample	Population (ACS 2020)
**Age**	Below 45 yrs	25.83	4.38	45.77
	45 yrs and above	74.18	95.62	54.23
**Gender**	Male	54.46	67.42	49.30
	Female	45.54	32.58	50.70
**Education**	No bachelor's degree	38.97	22.99	65.79
	At least bachelor's degree	61.03	77.01	34.21
**Income**	Under $20,000	4.69	0.36	10.91
	$20,000–$49,999	15.96	6.28	22.67
	$50,000–$74,999	22.07	12.56	17.42
	$75,000–$99,999	16.90	19.08	14.08
	$100,000–$149,999	19.25	25.47	18.30
	$150,000–$199,999	8.92	15.40	8.17
	$200,000 or more	12.21	20.85	8.45

Note: Chi-square goodness-of-fit tests were performed to compare both samples with the Minnesota population across gender, age, education, and household income. The general sample differs significantly in age, education, and household income but not in gender, while the lakeshore sample differs significantly across all characteristics. Minnesota population benchmarks are based on the American Community Survey (2020). Reported categories reflect the adult population aged 18 years and older.
