# Supplementary material for: Economic benefits of aquatic invasive species management in freshwater systems: A contingent valuation study
Source: PLoS One. 2026 Sep 16;21(9):e0346515. doi: 10.1371/journal.pone.0346515 (PMC13581201; doi:10.1371/journal.pone.0346515)
Supplement: Supplementary file 2 [file pone.0346515.s002.pdf]

S2 Table. Principal component analysis diagnostics and rotated component coefficients.

	**General**	**Lakeshore**
**Variable**	**Rotated coefficient**	**Rotated coefficient**
trust_DNR	0.57 (C1)	0.36 (C1)
trust_Uni	0.61 (C1)	0.51 (C1)
trust_EnvOrg	0.55 (C1)	0.48 (C1)
trust_lakeAssoc	—	0.38 (C1)
trust_LRadvocates	—	0.48 (C1)
trust_sportOrg	0.70 (C2)	0.69 (C2)
trust_stores	0.70 (C2)	0.68 (C2)
Diagnostics		
Bartlett's test of sphericity (p)	< 0.001	< 0.001
KMO measure of sampling adequacy	0.61	0.69
Cumulative variance explained (two components)	82.7%	65.0%
Cronbach's α, Component 1	0.84	0.79
Inter-item correlation, Component 2 (r)	0.81	0.67
N	287	1,198

Note: Values are rotated (orthogonal varimax) component coefficients, normalized so that the sum of squared coefficients in each column equals one (Stata default); coefficients below 0.30 are suppressed. Component 1 represents trust in public, research, and conservation entities; Component 2 represents trust in commercial and sporting entities. Component 1 comprises three items (general) and five items (lakeshore), for which we report Cronbach's α; Component 2 comprises two items in both samples, for which we report the inter-item correlation. PCA used all observations with non-missing trust responses. N = number of observations.
