# Supplementary material for: Economic benefits of aquatic invasive species management in freshwater systems: A contingent valuation study
Source: PLoS One. 2026 Sep 16;21(9):e0346515. doi: 10.1371/journal.pone.0346515 (PMC13581201; doi:10.1371/journal.pone.0346515)
Supplement: Supplementary file 3 [file pone.0346515.s003.pdf]

S3 Table. Respondents' agreement with statements reflecting ecocentric and anthropocentric values.

	General				Lakeshore			
**Statement**	**Mean**	**SD**	**% Agree**	**N**	**Mean**	**SD**	**% Agree**	**N**
Fisheries are valuable only if they produce jobs and income for people	1.957	1.025	8.920	209	1.743	1.005	6.161	844
Humans are no more important than other parts of nature	2.887	1.362	39.437	212	2.751	1.380	32.701	843
Fish are valuable only if people get to use them in some way	1.816	1.030	8.920	212	1.650	0.928	4.858	843
Nature's primary value is to provide things that are useful to people	1.867	1.087	10.798	211	1.705	1.011	7.701	843
Protecting the environment is more important than providing fishing opportunities	3.630	1.202	61.033	211	3.723	1.229	62.915	839
Humans are meant to rule over the rest of nature	2.048	1.263	17.371	210	2.023	1.200	15.047	843
Fish have as much right to exist as people	3.517	1.292	52.113	207	3.473	1.304	52.488	842
Fish are primarily valuable as food for people	2.623	1.184	25.352	207	2.335	1.060	15.758	841
Humans have a right to change the natural environment to suit their needs	2.014	1.161	14.554	211	1.897	1.086	12.204	842
Management should focus on doing what is best for nature instead of what is best for people	3.374	1.158	49.765	211	3.388	1.167	47.630	842

Note: SD = standard deviation. N = number of observations. % Agree is the percentage of respondents who either somewhat or strongly agreed with the statement. Because this portion of the dataset was not cleaned for modeling purposes, the number of observations varies slightly across items and samples.
