# Supplementary material for: Economic benefits of aquatic invasive species management in freshwater systems: A contingent valuation study
Source: PLoS One. 2026 Sep 16;21(9):e0346515. doi: 10.1371/journal.pone.0346515 (PMC13581201; doi:10.1371/journal.pone.0346515)
Supplement: Supplementary file 4 [file pone.0346515.s004.pdf]

S4 Table. Estimates from the probit model for the general resident survey.

	Unweighted			Weighted		
**Variables**	**(1)**	**(2)**	**(3)**	**(4)**	**(5)**	**(6)**
bid	-0.006*	-0.006**	-0.006**	-0.007*	-0.007**	-0.005
	(0.003)	(0.003)	(0.003)	(0.004)	(0.004)	(0.004)
age	-0.199	-	-	0.087	-	-
	(0.227)	-	-	(0.286)	-	-
female	-0.185	-	-	-0.075	-	-
	(0.208)	-	-	(0.242)	-	-
educ	-0.005	-	-	0.069	-	-
	(0.235)	-	-	(0.285)	-	-
income	0.062	-	-	0.020	-	-
	(0.063)	-	-	(0.072)	-	-
visit freq	0.115	-	-	0.144	-	-
	(0.094)	-	-	(0.115)	-	-
risk percep	0.261	0.292*	-	0.642***	0.562***	-
	(0.199)	(0.150)	-	(0.218)	(0.153)	-
trustscore1	0.248***	0.248***	-	0.256***	0.285***	-
	(0.081)	(0.073)	-	(0.088)	(0.084)	-
trustscore2	-0.090	-0.117*	-	-0.124	-0.176**	-
	(0.076)	(0.069)	-	(0.095)	(0.083)	-
fishing	-0.222	-	-	-0.416	-	-
	(0.253)	-	-	(0.303)	-	-
hiking	0.270	0.334*	-	0.401	0.509**	-
	(0.213)	(0.191)	-	(0.255)	(0.236)	-
motorboating	0.277	0.309	-	0.726**	0.648**	-
	(0.242)	(0.210)	-	(0.290)	(0.262)	-
AIS concern	0.153	-	-	0.009	-	-
	(0.160)	-	-	(0.183)	-	-
AIS knowledge	-0.107	-	-	-0.073	-	-
	(0.143)	-	-	(0.158)	-	-
lakeshore	-0.148	-	-	-0.168	-	-
	(0.250)	-	-	(0.307)	-	-
constant	-1.125	-0.886	0.433**	-2.675***	-2.034***	0.262
	(0.831)	(0.592)	(0.195)	(0.994)	(0.613)	(0.235)
N	213	213	213	213	213	213
Log Lik.	-124.938	-128.448	-145.150	-114.056	-116.461	-146.370
Pseudo R^2	0.151	0.127	0.013	0.227	0.211	0.009
AIC	281.876	270.896	294.301	260.112	246.921	296.740
BIC	335.656	294.425	301.023	313.892	270.451	303.462

Note: AIC = Akaike information criterion. BIC = Bayesian information criterion. Log Lik. = log likelihood. N = number of observations. Robust standard errors are provided in parentheses. * $p < 0.10$, ** $p < 0.05$, *** $p < 0.01$.
