# Supplementary material for: Economic benefits of aquatic invasive species management in freshwater systems: A contingent valuation study
Source: PLoS One. 2026 Sep 16;21(9):e0346515. doi: 10.1371/journal.pone.0346515 (PMC13581201; doi:10.1371/journal.pone.0346515)
Supplement: Supplementary file 5 [file pone.0346515.s005.pdf]

S5 Table. Spearman's rank correlation matrix for general resident survey.

Variables	bid	age	female	educ	income	visit freq	risk percep	trust-score1	trust-score2	fishing	hiking	motor-boating	knowledge	concern	lake-shore
bid	1.000														
age	-0.022	1.000													
female	0.045	0.044	1.000												
educ	0.003	-0.230	0.073	1.000											
income	-0.074	-0.174	-0.059	0.428	1.000										
visit freq	0.021	-0.137	-0.151	0.152	0.215	1.000									
risk percep	-0.071	0.113	0.106	-0.103	-0.145	0.058	1.000								
trustscore1	-0.073	0.086	0.289	0.138	-0.051	0.049	0.131	1.000							
trustscore2	-0.061	0.137	-0.170	-0.226	-0.102	-0.094	0.029	0.038	1.000						
fishing	-0.104	0.103	-0.333	-0.261	0.043	0.284	0.108	-0.087	0.269	1.000					
hiking	0.053	-0.088	0.151	0.282	0.060	0.360	0.047	0.151	-0.271	-0.061	1.000				
motorboating	0.116	-0.012	-0.175	0.051	0.146	0.315	0.015	-0.084	0.114	0.392	0.009	1.000			
knowledge	0.060	0.087	-0.263	-0.083	0.004	0.288	0.310	-0.008	0.105	0.374	0.124	0.234	1.000		
concern	-0.072	0.269	-0.054	-0.107	-0.186	0.085	0.666	0.085	0.171	0.239	-0.036	0.048	0.328	1.000	
lakeshore	0.027	0.099	-0.128	0.011	0.242	0.255	0.099	-0.061	0.059	0.322	-0.029	0.244	0.330	0.145	1.000

Note: knowledge = AIS knowledge. concern = AIS concern. The shaded cells indicate correlation coefficients that are statistically significant at the 5% level.
