# Supplementary material for: Economic benefits of aquatic invasive species management in freshwater systems: A contingent valuation study
Source: PLoS One. 2026 Sep 16;21(9):e0346515. doi: 10.1371/journal.pone.0346515 (PMC13581201; doi:10.1371/journal.pone.0346515)
Supplement: Supplementary file 6 [file pone.0346515.s006.pdf]

S6 Table. Percentage of yes and total sample in each bid amount after eliminating protest and 'DK' categories from the sample.

	General		Lakeshore	
**Bid**	**% Yes**	**n**	**% Yes**	**n**
20	91.18	34	95.36	151
40	90.00	30	96.55	145
60	88.89	18	98.29	124
80	68.42	19	97.04	135
100	90.00	30	91.89	148
